# Prognostic Value of the Pretreatment Systemic Immune-Inflammation Index in Patients with Colorectal Cancer

**DOI:** 10.1155/2020/8781674

**Published:** 2020-11-20

**Authors:** Jing Li, Jingjing Shao, Xunlei Zhang, Xin Chen, Wenjing Zhao, Hongyan Qian, Xiaopeng Cui, Xiaohui Jiang

**Affiliations:** ^1^Cancer Research Center Nantong, Nantong Tumor Hospital & Affiliated Tumor Hospital of Nantong University, Nantong, China; ^2^Department of Oncology, Nantong Tumor Hospital & Affiliated Tumor Hospital of Nantong University, Nantong, China; ^3^Department of General Surgery, Nantong Tumor Hospital & Affiliated Tumor Hospital of Nantong University, China; ^4^Department of Gastrointestinal Surgery, Nantong University Affiliated Hospital, Nantong, Jiangsu, China

## Abstract

**Background:**

Multiple studies have reported the significance of the systemic immune-inflammation index (SII) in the prognosis of colorectal cancer (CRC), but no consensus has yet been reached. The purpose of this study was to systematically assess the prognostic value of SII in patients with CRC.

**Materials and Methods:**

We performed a systematic literature search in PubMed, Embase, and the Cochrane Library for eligible studies. The correlation between pretreatment SII and overall survival (OS), disease-free survival (DFS), and progression-free survival (PFS) in CRC patients was evaluated by combining the hazard ratio (HR) and 95% confidence interval (CI).

**Results:**

Twelve studies involving 3919 patients were included. Comprehensive analysis results showed that high SII indicated poor OS in CRC patients (HR = 1.777, 95% CI: 1.328-2.376). Compared with patients with low SII values, patients with high SII had lower PFS (HR = 1.658, 95% CI: 1.189-2.311). Subgroup analysis further verified the above results.

**Conclusions:**

SII may be a noninvasive and powerful tool for predicting survival outcomes in CRC patients. However, more well-designed studies are needed to validate our findings.

## 1. Introduction

The latest epidemiological survey data shows that the incidence of colorectal cancer (CRC) ranks third and is the second leading cause of cancer-related deaths in the world [1]. Despite the progress made in early diagnosis and multidisciplinary treatment, the prognosis of patients is still poor, and the 5-year survival rate of CRC patients remains at around 65% [2]. Therefore, it is necessary to identify accurate and reliable prognostic markers for CRC patients.

In recent years, as a noninvasive detection method, serum biomarkers have attracted more and more attention because of their simple operation and large predictive potential [3]. Several studies have shown that the systemic immune-inflammation index (SII) is related to the prognosis of malignant tumors [4]. SII is a promising biomarker based on inflammation, mainly calculated from the counts of lymphocytes, neutrophils, and platelets, and each parameter can be easily measured from venous blood samples [5, 6]. As a promising prognostic marker for CRC, SII has attracted extensive attention [6–14]. Several studies have shown that SII can be used as a valuable predictor of different treatment methods for CRC. Chen et al. indicated that the prognostic value of SII was determined to be superior to those of NLR and PLR after radical resection of CRC for the first time [6]. Other studies have also confirmed the prognostic value of SII to predict the efficacy of bevacizumab in mCRC [7, 9]. With the successful application of immune checkpoint inhibitors (ICIs) in a variety of tumors, host immune responses, especially enhanced lymphocyte responses, have become the focus of recent research [15, 16]. Xie et al. reported that SII was an independent predictor of the prognosis of mCRC and was associated with lymphocyte response to tumors [11]. Moreover, this study also suggests that systemic inflammation reflected by SII may be related to the tumor and tumor microenvironment [11]. However, other research has achieved inconsistent or even contradictory results in terms of the prognostic value [17–19]. In addition, the SII cut-off values used in the results of these studies are not the same. So far, the impact of SII on the prognosis of CRC patients has not been systematically studied.

In this study, we performed this meta-analysis to review and summarize all available data to determine the effect of SII on the prognosis of CRC and its implications for clinicopathological parameters.

## 2. Materials and Methods

This meta-analysis was conducted according to the Preferred Reporting Items for Systematic Reviews and Meta-Analyses (PRISMA) guidelines [20], and a PRISMA checklist is provided in the supplementary result.

### 2.1. Search Strategy and Study Selection

We performed a systematic literature search in PubMed, Embase, and the Cochrane Library for eligible studies updated in May 1, 2020. The search strategies for this study included the following terms: “systemic immunoinflammatory index” or “neutrophil platelet/lymphocyte” or “SII” and “cancer” OR “neoplasm” OR “tumor” OR “carcinoma.” And we also manually screened the retrieved references to find the relevant potential literature. Detailed information of the search strategy can be found in the supplementary material.

### 2.2. Inclusion and Exclusion Criteria

The inclusion criteria were as follows: (1) articles exploring the relationship between SII and the prognosis of CRC; (2) neutrophil, platelet, and lymphocyte counts measured before treatment, including surgery, neoadjuvant chemoradiotherapy, and chemoradiotherapy; (3) data including overall survival (OS), disease-free survival (DFS), or progression-free survival (PFS) and risk ratios (HRs), as well as the corresponding 95% confidence interval (95% CI); (4) patient size greater than 50; (5) all patients included in the study being divided into two groups based on counting scores; and (6) all publications with full text written in English.

Excluded articles include articles that are not specific to CRC or that involve animals or reviews, meta-analyses, poster sessions, conference abstracts, etc.

### 2.3. Data Extraction and Quality Assessment

Information was extracted from the included studies: first author name, year of publication, country, ethnicity, study type, study period, sample size, cut-off value, treatment, clinicopathological factors, follow-up time, prognostic indicators, and HRs and their corresponding 95% CIs. Data collection uses Excel forms (Microsoft Corporation). The quality assessment of the included studies was conducted by two independent researchers (Jing Li and Jingjing Shao) according to the Newcastle-Ottawa quality assessment scale (NOS) [21]. A study with a score of 6 or more was defined as a high-quality study.

### 2.4. Statistical Analysis

Stata 14.0 software (Stata, College Station, Texas) was used to analyze the extracted data and combine HR. The Higgins *I*^2^ statistic and Cochran's *Q* test were used to assess the heterogeneity between studies. If *P* < 0.1 and/or *I*^2^ > 50%, it is defined as significant heterogeneity and a random effects model is used; otherwise, a fixed effects model is used [22]. Potential publication bias was determined by funnel plots and Begg's test/Egger's test. The robustness of the combined data was evaluated by sensitivity analysis. *P* < 0.05 was considered statistically significant.

## 3. Results

### 3.1. Study Characteristics

**Figure 1:**
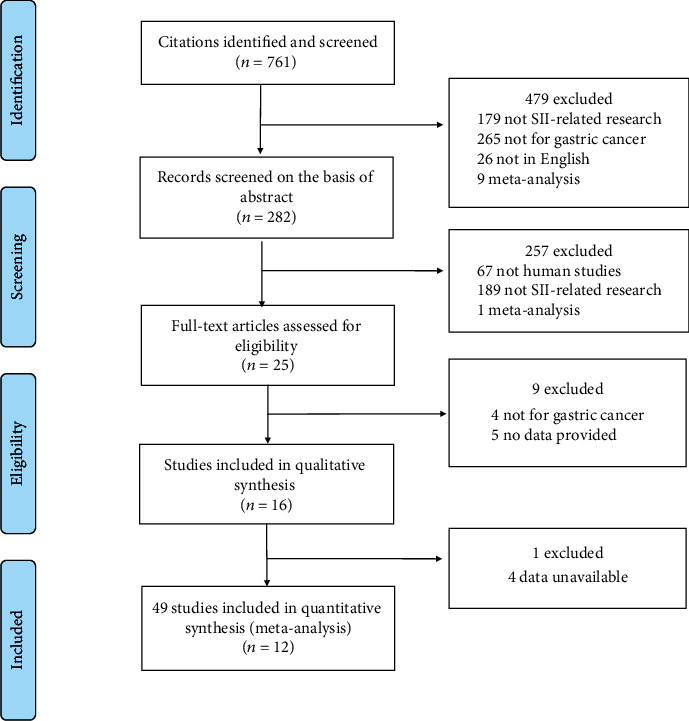
The flowchart for literature screening.

As shown in Figure 1, a total of 761 articles were obtained. After eliminating duplicates and screening for titles and abstracts, 16 studies were eligible for a comprehensive test. Finally, a total of 12 articles involving 3919 patients were included. Details of these studies are shown in Table 1. Most of the included studies are retrospective studies, there is only one prospective study, these articles were published in 2016-2019, and the sample size was between 95 and 1383. One of the studies was conducted in Italy [7], and the other studies were conducted in China [6, 8–14, 17–19]. The SII cut-off values ranged from 340 to 1505.

### 3.2. Relationship between SII and Survival Outcomes in CRC

**Figure 2:**
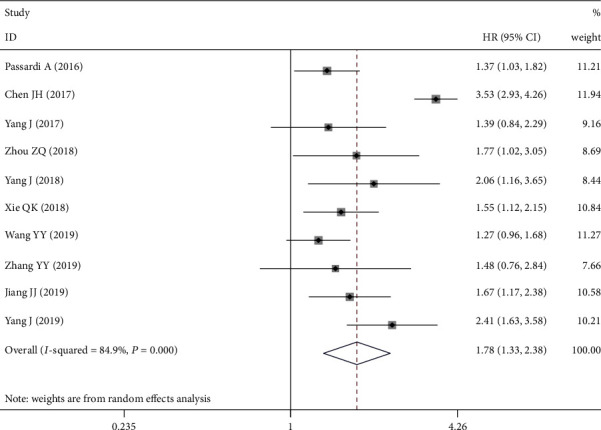
Forest plot for the association between SII and overall survival. SII: systemic immune-inflammation index.

**Figure 3:**
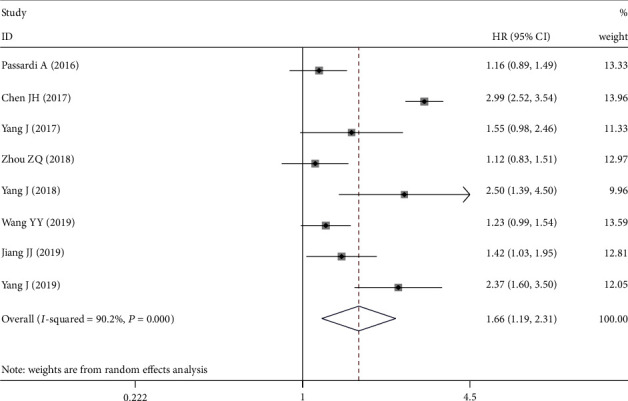
Forest plot for the association between SII and disease-free survival/progression-free survival. SII: systemic immune-inflammation index.

A total of 10 cohort studies were combined with 3619 cases to explore the association between SII and OS in CRC. Significant heterogeneity was observed between studies (*I*^2^ = 84.9%, *P*_H_ = 0.000), so the random effects model was chosen. The combined HR was 1.777, and the 95% CI was 1.328~2.376. The results indicated that higher SII was a prognostic factor for poor OS in CRC patients (Figure 2). As shown in Figure 3, eight studies reported the relationship between SII and DFS/PFS. As obvious heterogeneity between these studies was observed, the random effects model was used (*I*^2^ = 90.2%, *P*_H_ = 0.000). Combined analysis shows that SII is an independent predictor of DFS/PFS in CRC patients.

### 3.3. Subgroup Analysis

To further study the prognostic value of SII, we performed subgroup analysis stratified by ethnicity, treatment, cut-off value, and sample size (Table 2). Our results showed that high SII predicted poor OS for all subgroups. The predictive power of SII was also strong in nonsurgery patients and patients with a cut-off value above 500.

Table 3 shows the relationship between SII and clinicopathological parameters. The results showed that high SII was significantly correlated with higher ECOG performance status and poor T stage, but not with primary tumor site, tumor differentiation, and chemotherapy.

### 3.4. Publication Bias

**Figure 4:**
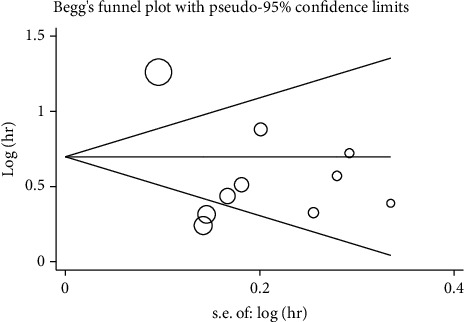
Begg' s funnel plot for the publication bias test.

Figure 4 shows that there was no obvious publication deviation in this meta-analysis, and the funnel plot of Begg (*P* = 0.283) was symmetrical, and the *P* value of Egger's test was 0.142.

### 3.5. Sensitivity Analysis

**Figure 5:**
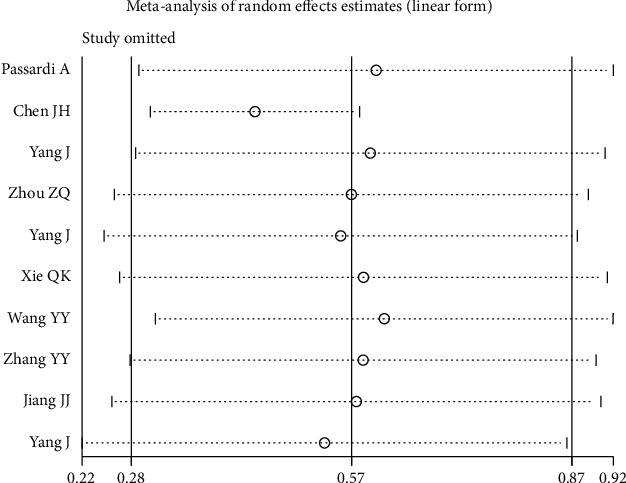
Sensitivity analysis for the association between SII and overall survival. SII: systemic immune-inflammation index.

Sensitivity analysis was performed to assess the potential impact of individual studies on the combined results, which showed that the combined results remained stable after the exclusion of any study (Figure 5).

## 4. Discussion

As is known to all, chronic inflammation is one of the important mechanisms for the development of colon cancer, and the important role of cancer-related inflammation in the development and progression of cancer has been a hot topic of research in recent years [23]. The link between chronic inflammation and cancer is clear, with about 20% of human cancers associated with precancerous inflammation [24]. Inflammatory bowel disease (IBD) patients with elevated inflammatory markers had a significant 2- to 8-fold increased risk of CRC [25]. However, in the study of primary CRC without intestinal inflammation, it was found that the tumor tissue was still infiltrated by inflammatory cells, and the expression level of inflammatory cytokines was significantly increased. And inflammatory bowel disease obviously increases the risk of CRC, so inflammation is highly related to the occurrence and development of CRC [26]. Due to the convenience, low cost, and rapid detection of systemic inflammatory markers, research on inflammatory biomarkers for tumor prognosis has been increasing recently. These inflammation indicators derived from white blood cells, such as NLR, PLR, and SII, have been proven to have important clinical significance in many types of cancer, such as CRC, gastric cancer, and hepatocellular carcinoma [8, 27]. As an important inflammation-related indicator, NLR has been reported as a predictive marker for various malignancies, including CRC [27]. SII, calculated as neutrophils × platelets/lymphocytes, is a combination of PLR and NLR, and it had been suggested that SII may perform better as a prognostic indicator than NLR [4]. As a more objective tumor marker, SII could reflect both the inflammatory response and the immune response of the host.

Several studies suggest that SII can be used as a predictor of prognosis in CRC patients [6–14]. However, the exact underlying mechanism is still poorly understood. In recent years, some preclinical data have shown that cells involved in the bone microenvironment and immune system can promote tumor growth and development. The bone niche represents a sanctuary for cancer cells to resist anticancer treatments [28]. And hematopoiesis occurs in the bone and is guaranteed by the bone niche, by detecting various cells in the blood; the bone microenvironment and immune microenvironment can be well reflected [29]. SII is a comprehensive indicator composed of myeloid-derived neutrophils, lymphocytes, and platelets, which can reflect the host's immune response to tumor cells. Neutrophils may be indicators of acute and chronic inflammation and play a role in the development and progression of tumors [30]. It not only changes the microenvironment of tumors through external pathways but also secretes some inflammatory mediators through internal pathways to promote tumor cell proliferation, invasion, and metastasis to lymph nodes or distant organs [31]. Conversely, lymphocytes in the blood of tumor patients are usually reduced, which may help tumor cells to escape immune surveillance and avoid damage caused by cytotoxic T cell immune responses [32]. In addition, in a variety of gastrointestinal cancers, lymphopenia has been reported to be associated with poor cancer survival [33]. In addition, studies had found that patients with malignant tumors are often accompanied by increased platelets, which were found to play an important role in tumor progression and metastasis. On the one hand, activated platelets induce the formation of the optimal metastatic environment, facilitating the epithelial-mesenchymal transformation of tumor cells in circulation; on the other hand, they help tumor cells escape the surveillance of the host immune system [34]. Cancer-related thrombocytosis can suppress host immunity by subverting anticancer T cell immunity [35]. Therefore, high levels of neutrophils and platelets, and low levels of lymphocytes, could be reflected in high levels of SII, which all indicated a weak immune response but a strong inflammatory response in patients. It may be associated with tumor invasion and metastasis, resulting in poor survival of patients [27].

In this meta-analysis, a total of 12 published articles were included, including 3919 cases. By analysis, we found that high levels of SII represent poor prognosis in CRC patients. In addition, we performed a subgroup analysis to assess the prognostic significance of SII. Our results showed that high SII predicted poor OS for all subgroups. The predictive power of SII was also strong in nonsurgery patients and patients with a cut-off value above 500. In addition, we found a significant association between high SII values and poor DFS/PFS in CRC. In summary, SII might be considered a prognostic marker with great clinical and practical value for patients with CRC.

Our study was associated with several limitations. First, the inclusion criteria for this meta-analysis were limited to the studies published in English. And some studies without sufficient data were excluded. Thus, publication or data availability bias may exist. Second, almost all the included studies were retrospective, with only one prospective study, and the patients included were all but composed of an Asian cohort, leading to the possibility of greater susceptibility to bias. However, no significant publication bias occurred based on the result in the asymmetry of the funnel plot, thus maintaining the substantial consistency among the results. Third, there was considerable heterogeneity when pooling HRs for OS results. Subgroup analysis showed that the cut-off values in the included studies were varied, which could lead to heterogeneity between studies. And the biological behavior and prognosis of CRC with different BRAF and KRAS mutants are different [36, 37]. It is expected that more studies will provide data to prove the prognostic value of SII in different BRAF and KRAS mutation states in CRC. Finally, most of the included studies have no validation cohort. Higher-quality studies are expected to assess the relationship more accurately between SII and CRC prognosis.

## 5. Conclusion

In conclusion, this meta-analysis demonstrates that high SII is significantly associated with poor clinical outcomes in CRC patients. As a valuable, noninvasive serological indicator, SII can be used to predict the prognosis of CRC patients. However, large-scale, prospective, and multicenter studies are needed to validate our findings.

## Figures and Tables

**Table 1:** Characteristics of all the studies included in the meta-analysis.

Author	Year	Country	Ethnicity	Treatment	Follow-up (month)	Cut-off	Study period	Patients (*n*)	Survival analysis	NOS score
Passardi	2016	Italy	Europe	No surgery	36 (1-65)	730	2007-2012	289	OS/PFS	8
Yang	2017	China	Asian	No surgery	40 (12-72)	460.66	2009-2015	95	OS/PFS	7
Chen	2017	China	Asian	With surgery	>60	340	1994-2010	1383	OS/PFS	8
Zhou	2018	China	Asian	With surgery	21.72 (2.11-118.72)	385.91	2007-2015	516	OS/PFS	8
Yang	2018	China	Asian	With surgery	37.0 (16.2-93.3)	437.72	2010-2015	98	OS/PFS	7
Tao	2018	China	Asian	With surgery	NA	667.75	2011-2013	118	NA	6
Xie	2018	China	Asian	With surgery	26.7 (1.1-92.4)	649.45	2009-2014	240	OS	6
Wang	2019	China	Asian	With surgery	28 (19-46)	517	2002-2016	452	OS/DFS	8
Zhang	2019	China	Asian	With surgery	NA	NA	2010-2013	224	OS	6
Lu	2019	China	Asian	With surgery	NA	1505	2010-2017	182	NA	6
Jiang	2019	China	Asian	No surgery	33.2 (2.6-94.5)	660.55	2010-2017	102	OS/PFS	7
Yang	2019	China	Asian	No surgery	23.9 (12-87)	534.94	2009-2015	220	OS/PFS	7

OS: overall survival; PFS: progression-free survival; DFS: disease-free survival; NA: not available; NOS: Newcastle-Ottawa quality assessment scale.

**Table 2:** Subgroup analyses.

	Factors	No. of studies	No. of patients	Effects model	HR (95% CI)	*P*	Heterogeneity	
							*I* ^2^	*P*_H_
OS	Overall	10	3619	Random	1.777 (1.328-2.376)	<0.001	84.90%	0
	Ethnicity							
	Europe	1	289		1.370 (1.030-1.820)	<0.001		
	Asia	9	3330	Random	1.835 (1.342-2.509)	<0.001	84.40%	0
	Treatment							
	No surgery	4	706	Fixed	1.626 (1.357-1.947)	<0.001	46.90%	0.13
	With surgery	5	2913	Fixed	2.244 (1.971-2.555)	<0.001	89.10%	0
	Cut-off							
	≤500	4	2092	Fixed	2.895 (2.465-3.399)	<0.001	82.40%	0.001
	>500	5	1303	Fixed	1.529 (1.326-1.764)	<0.001	47.60%	0.106
	Sample size							
	≤200	3	295	Fixed	1.658 (1.280-2.147)	<0.001	0.00%	0.593
	>200	7	3324	Fixed	2.089 (1.862-2.345)	<0.001	89.20%	0
PFS	Overall	8	3155	Random	1.658 (1.189-2.311)	<0.01	90.20%	0

CI: confidence interval; HR: hazard ratio; OS: overall survival; PFS: progression-free survival.

**Table 3:** Associations between SII and clinicopathological parameters.

Variable	No. of studies	No. of patients	Effects model	OR (95% CI)	*P*	Heterogeneity	
						*I* ^2^	*P*_H_
ECOG performance status (0 vs. 1-2)	3	607	Fixed	1.212 (1.097-1.340)	<0.001	0.00%	0.732
Tumor location (colon vs. rectum)	5	884	Fixed	0.928 (0.839-1.027)	0.149	40.80%	0.15
Tumor differentiation (moderate/high vs. poor)	6	2096	Random	1.058 (0.953-1.175)	0.291	64.90%	0.014
T (0-2 vs. 3)	3	1663	Fixed	1.651 (1.380-1.976)	<0.001	32.10%	0.229
Chemotherapy (no vs. yes)	3	486	Fixed	1.139 (0.962-1.350)	0.132	2.70%	0.358

ECOG: Eastern Cooperative Oncology Group; SII: systemic immune-inflammation index; OR: odds ratio; CI: confidence interval.

## Data Availability

The data used in this meta-analysis can be obtained from the corresponding authors upon request.

## Supplementary Materials

Supplementary Materials Supplementary Table 1: search strategies—the detailed search strategy (using PubMed as an example) was listed.

## References

[1] Bray F., Ferlay J., Soerjomataram I., Siegel R. L., Torre L. A., Jemal A. (2018). Global cancer statistics 2018: GLOBOCAN estimates of incidence and mortality worldwide for 36 cancers in 185 countries. *CA: a Cancer Journal for Clinicians*.

[2] Miller K. D., Siegel R. L., Lin C. C. (2016). Cancer treatment and survivorship statistics, 2016. *CA: a Cancer Journal for Clinicians*.

[3] Khan F., Vogel R. I., Diep G. K., Tuttle T. M., Lou E. (2016). Prognostic factors for survival in advanced appendiceal cancers. *Cancer Biomarkers*.

[4] Geng Y., Shao Y., Zhu D. (2016). Systemic immune-inflammation index predicts prognosis of patients with esophageal squamous cell carcinoma: a propensity score-matched analysis. *Scientific Reports*.

[5] Hu B., Yang X. R., Xu Y. (2014). Systemic immune-inflammation index predicts prognosis of patients after curative resection for hepatocellular carcinoma. *Clinical Cancer Research*.

[6] Chen J. H., Zhai E. T., Yuan Y. J. (2017). Systemic immune-inflammation index for predicting prognosis of colorectal cancer. *World Journal of Gastroenterology*.

[7] Passardi A., Scarpi E., Cavanna L. (2016). Inflammatory indexes as predictors of prognosis and bevacizumab efficacy in patients with metastatic colorectal cancer. *Oncotarget*.

[8] Zhou Z. Q., Pang S., Yu X. C. (2018). Predictive values of postoperative and dynamic changes of inflammation indexes in survival of patients with resected colorectal cancer. *Current Medical Science*.

[9] Yang J., Xu H., Guo X. (2018). Pretreatment inflammatory indexes as prognostic predictors for survival in colorectal cancer patients receiving neoadjuvant chemoradiotherapy. *Scientific Reports*.

[10] Tao M. Y., Wang Z. H., Zhang M. H. (2018). Prognostic value of the systematic immune-inflammation index among patients with operable colon cancer: a retrospective study. *Medicine (Baltimore)*.

[11] Xie Q. K., Chen P., Hu W. M. (2018). The systemic immune-inflammation index is an independent predictor of survival for metastatic colorectal cancer and its association with the lymphocytic response to the tumor. *Journal of Translational Medicine*.

[12] Lu Y., Xin D., Wang F. (2019). Predictive significance of preoperative systemic immune-inflammation index determination in postoperative liver metastasis of colorectal cancer. *Oncotargets and Therapy*.

[13] Jiang J., Ma T., Xi W. (2019). Pre-treatment inflammatory biomarkers predict early treatment response and favorable survival in patients with metastatic colorectal cancer who underwent first line cetuximab plus chemotherapy. *Cancer Management and Research*.

[14] Yang J., Guo X., Wu T., Niu K., Ma X. (2019). Prognostic significance of inflammation-based indexes in patients with stage III/IV colorectal cancer after adjuvant chemoradiotherapy. *Medicine (Baltimore)*.

[15] Basile D., Garattini S. K., Bonotto M. (2017). Immunotherapy for colorectal cancer: where are we heading?. *Expert Opinion on Biological Therapy*.

[16] Garon E. B., Rizvi N. A., Hui R. (2015). Pembrolizumab for the treatment of non-small-cell lung cancer. *The New England Journal of Medicine*.

[17] Wang Y. Y., Liu Z. Z., Xu D., Liu M., Wang K., Xing B. C. (2019). Fibrinogen-albumin ratio index (FARI): a more promising inflammation-based prognostic marker for patients undergoing hepatectomy for colorectal liver metastases. *Annals of Surgical Oncology*.

[18] Zhang Y. Y., Li W. Q., Li Z. F. (2019). Higher levels of pre-operative peripheral lymphocyte count is a favorable prognostic factor for patients with stage I and II rectal cancer. *Frontiers in Oncology*.

[19] Yang J., Guo X., Wang M., Ma X., Ye X., Lin P. (2017). Pre-treatment inflammatory indexes as predictors of survival and cetuximab efficacy in metastatic colorectal cancer patients with wild-type RAS. *Scientific Reports*.

[20] Moher D., Liberati A., Tetzlaff J., Altman D. G., The PRISMA Group (2009). Preferred reporting items for systematic reviews and meta-analyses: the PRISMA statement. *PLoS Medicine*.

[21] Stang A. (2010). Critical evaluation of the Newcastle-Ottawa scale for the assessment of the quality of nonrandomized studies in meta-analyses. *European Journal of Epidemiology*.

[22] Barili F., Parolari A., Kappetein P. A., Freemantle N. (2018). Statistical primer: heterogeneity, random- or fixed-effects model analyses?. *Interactive Cardiovascular and Thoracic Surgery*.

[23] Diakos C. I., Charles K. A., McMillan D. C., Clarke S. J. (2014). Cancer-related inflammation and treatment effectiveness. *The Lancet Oncology*.

[24] Grivennikov S. I., Greten F. R., Karin M. (2010). Immunity, inflammation, and cancer. *Cell*.

[25] Terzic J., Grivennikov S., Karin E., Karin M. (2010). Inflammation and colon cancer. *Gastroenterology*.

[26] Ning Y., Manegold P. C., Hong Y. K. (2011). Interleukin-8 is associated with proliferation, migration, angiogenesis and chemosensitivity in vitro and in vivo in colon cancer cell line models. *International Journal of Cancer*.

[27] Zhong J. H., Huang D. H., Chen Z. Y. (2017). Prognostic role of systemic immune-inflammation index in solid tumors: a systematic review and meta-analysis. *Oncotarget*.

[28] Gnoni A., Brunetti O., Longo V. (2020). Immune system and bone microenvironment: rationale for targeted cancer therapies. *Oncotarget*.

[29] Schreiber R. D., Old L. J., Smyth M. J. (2011). Cancer immunoediting: integrating immunity's roles in cancer suppression and promotion. *Science*.

[30] Tan K. W., Chong S. Z., Wong F. H. (2013). Neutrophils contribute to inflammatory lymphangiogenesis by increasing VEGF-A bioavailability and secreting VEGF-D. *Blood*.

[31] Felix K., Gaida M. M. (2016). Neutrophil-derived proteases in the microenvironment of pancreatic cancer-active players in tumor progression. *International Journal of Biological Sciences*.

[32] Quigley D. A., Kristensen V. (2015). Predicting prognosis and therapeutic response from interactions between lymphocytes and tumor cells. *Molecular Oncology*.

[33] Saito H., Kono Y., Murakami Y. (2019). Prognostic significance of pre- and postoperative lymphocyte counts in patients with gastric cancer. *Digestive Surgery*.

[34] Coupland L. A., Parish C. R. (2014). Platelets, selectins, and the control of tumor metastasis. *Seminars in Oncology*.

[35] Rachidi S., Metelli A., Riesenberg B. (2017). Platelets subvert T cell immunity against cancer via GARP-TGF*β* axis. *Science Immunology*.

[36] Zlobec I., Bihl M. P., Schwarb H., Terracciano L., Lugli A. (2010). Clinicopathological and protein characterization of BRAF- and K-RAS-mutated colorectal cancer and implications for prognosis. *International Journal of Cancer*.

[37] Santini D., Spoto C., Loupakis F. (2010). High concordance of BRAF status between primary colorectal tumours and related metastatic sites: implications for clinical practice. *Annals of Oncology*.

