# Supplementary material for: Prognostic Value of the Pretreatment Systemic Immune-Inflammation Index in Patients with Colorectal Cancer
Source: Gastroenterol Res Pract. 2020 Nov 20;2020:8781674. doi: 10.1155/2020/8781674 (PMC7700049; doi:10.1155/2020/8781674)
Supplement: Supplementary Materials — Supplementary Table 1: search strategies—the detailed search strategy (using PubMed as an example) was listed. [file 8781674.f1.docx]

Supplementary Table. 1 Search Strategies

The detailed search strategy (using PubMed as an example) was listed below:

Relevant studies were obtained from PUBMED up to May 1, 2020

| 1. "neoplasms"[Mesh] |
| --- |
| 2. “cancer” [All Fields] |
| 3. “tumor” [All Fields] |
| 4. “carcinoma” [All Fields] |
| 5. 1 OR 2 OR 3 OR 4 |
| 6. " systemic immunoinflammatory index "[ All Fields] |
| 7. " neutrophil platelet/lymphocyte "[ All Fields] |
| 8. "SII" [All Fields] |
| 9. 6 OR 7 OR 8 |
| 10. 5 AND 9 |
